# Prognostic value of circulating microRNAs in upper tract urinary carcinoma

**DOI:** 10.18632/oncotarget.24672

**Published:** 2018-03-30

**Authors:** Ruth Montalbo, Laura Izquierdo, Mercedes Ingelmo-Torres, Juan José Lozano, David Capitán, Antonio Alcaraz, Lourdes Mengual

**Affiliations:** ^1^ Department and Laboratory of Urology, Hospital Clínic, Institut d'Investigacions Biomèdiques August Pi i Sunyer, Universitat de Barcelona, Barcelona, Spain; ^2^ CIBERehd, Plataforma de Bioinformática, Centro de Investigación Biomédica en red de Enfermedades Hepáticas y Digestivas, Universidad de Barcelona, Barcelona, Spain

**Keywords:** biomarkers, microRNA, serum, tumour progression, upper urinary tract urothelial carcinoma

## Abstract

The identification of upper tract urinary carcinoma (UTUC) prognostic biomarkers is urgently needed to predict tumour progression. This study aimed to identify serum microRNAs (miRNAs) that may be useful as minimally invasive predictive biomarkers of tumour progression and survival in UTUC patients. To this end, 33 UTUC patients who underwent radical nephroureterectomy at the Hospital Clinic of Barcelona were prospectively included. Expression of 800 miRNAs was evaluated in serum samples from these patients using nCounter® miRNA Expression Assays. The study was divided into an initial discovery phase (n=12) and a validation phase (n=21). Cox regression analysis was used for survival analysis. The median follow-up (range) of the series was 42 months (9-100 months). In the discovery phase, 38 differentially expressed miRNAs were identified between progressing and non-progressing UTUC patients (p<0.05). Validation of these 38 miRNAs in an independent set of UTUC patients confirmed the differential expression in 18 of them (p<0.05). Cox Regression analysis showed miR-151b and pathological stage as significant prognostic factors for tumour progression (HR=0.33, p<0.001 and HR=2.62, p=0.006, respectively) and cancer specific survival (HR=0.25, p<0.001 and HR=3.98, p=0.003, respectively). Survival curves revealed that miR-151b is able to discriminate between two groups of UTUC patients with a highly significant different probability of tumour progression (p=0.006) and cancer specific survival (p=0.034). Although the data needs to be externally validated, miRNA analysis in serum appears to be a valuable prognostic tool in UTUC patients. Particularly, differential expression of miR-151b in serum may serve as a minimally invasive prognostic tool in UTUC.

## INTRODUCTION

Radical nephroureterectomy (RNU) is the accepted treatment for localized upper tract urinary carcinoma (UTUC) [1]. Pathological stage and tumour grade are the most established prognostic factors associated with tumour progression and patient survival, but they are insufficient to predict the individual outcome of UTUC patients [2]. More accurate knowledge regarding the biological behaviour of tumours would allow for tailored treatment schedules to be offered to patients (such as neodjuvant chemotherapy or early radical surgery), in an attempt to increase survival and decrease morbidity.

The rapid advance in the understanding of the molecular biology of UTUC has led to the appearance of promising new biomarkers such as microRNAs (miRNAs) [3–6]. miRNAs are an abundant class of newly identified endogenous non-protein-coding small RNAs with a 20-25 nucleotide length [7], which can negative-regulate protein expression of target genes. Recent studies show that several miRNAs are differentially expressed in different human cancers, which indicate that miRNAs may have a role in the carcinogenetic processes of numerous tumours [8–11]. Interestingly, these miRNA differences have also been observed in human biofluids [12, 13]. It has been recently demonstrated that circulating miRNAs in the blood stream are present in a stable and reproducible state [14]. Moreover, blood samples are easily acquired in a relatively non-invasive manner and miRNAs from blood can be readily detected [15]. Notably, the serum miRNA expression profile has been used as a fingerprint for various malignancies, including carcinomas of the urinary tract [13, 16]. In particular, diagnostic miRNAs in serum have been described for UTUC [17]. However, to the best of our knowledge, the prognostic global circulating miRNA patterns from UTUC patients have not been studied as yet.

In the present work, we aim to determine the differential miRNA expression patterns in serum of progressing and non-progressing UTUC patients in order to identify putative miRNAs that may be used as prognostic markers.

## RESULTS

### Clinical features

Median age (range) of the series was 70 (53-90) years. The median (range) follow-up of the cohort was 42 (9-100) months. Tumour progression was documented in 13 patients (39%). The median (range) time of tumour progression was 10 (2-46) months. Eleven patients (33%) died due to their UTUC. The median (range) time of cancer specific death was 24 (6-48) months. Two patients with tumour progression died from diseases other than UTUC.

### Discovery phase

**Figure 1:**
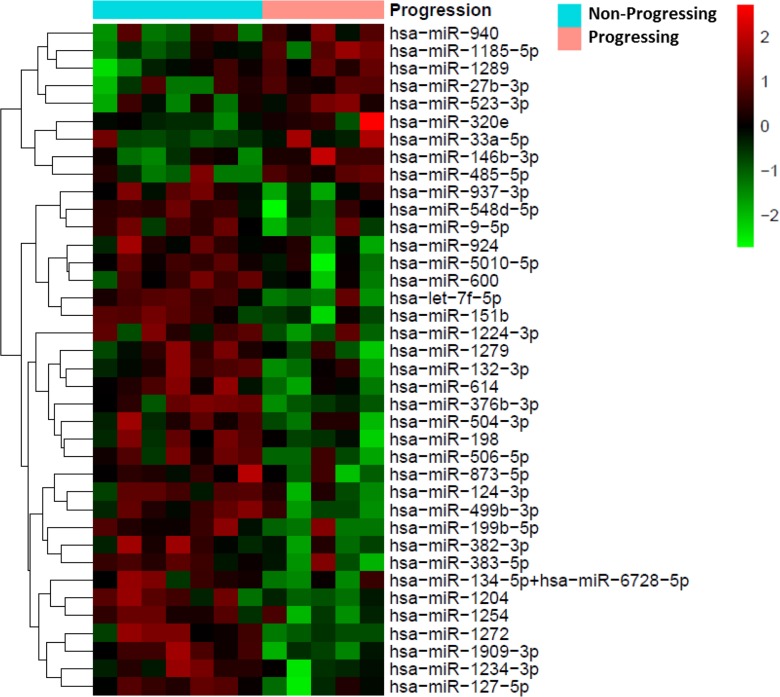
Heat map showing the 38 miRNAs differentially expressed between patients with progressing and non-progressing UTUC (N=12) Red pixels correspond to a greater abundance of miRNA in the serum samples, whereas green pixels indicate lower miRNA levels. Rows represent individual miRNAs and columns represent experimental samples.

Overall, 38 miRNAs were identified as differentially expressed between progressing (n=5) and non-progressing (n=7) UTUC patients. Of these, nine miRNAs were up-regulated and 29 down-regulated in progressing patients. A heat map based on the differentially expressed miRNAs between the two groups of UTUC patients is shown in Figure 1.

### Validation phase

All 38 miRNAs previously identified as differentially expressed were analyzed in an independent set of 21 UTUC serum samples (8 progressing and 13 non-progressing). Eighteen microRNAs remained differentially expressed between these two groups of UTUC patients; all of them were down-regulated in progressing patients (Table 1).

**Table 1:** List of the 18 differentially expressed miRNAs and their FC values in the validation phase

miRNA	p-value	FC	FDR (%)
hsa-miR-600	0.001	−2.32	2.07
hsa-miR-504-3p	0.002	−2.30	2.431
hsa-miR-1279	0.003	−2.29	2.712
hsa-miR-198	0.001	−2.28	2.141
hsa-miR-151b	0.003	−2.13	2.747
hsa-miR-499b-3p	0.010	−1.99	3.329
hsa-miR-937-3p	0.008	−1.94	3.199
hsa-miR-1909-3p	0.018	−1.87	3.995
hsa-miR-383-5p	0.031	−1.86	5.357
hsa-miR-376b-3p	0.024	−1.86	4.755
hsa-miR-924	0.039	−1.82	6.143
hsa-miR-1254	0.016	−1.82	3.846
hsa-miR-1204	0.041	−1.80	6.332
hsa-miR-1272	0.020	−1.77	4.185
hsa-miR-132-3p	0.032	−1.76	5.416
hsa-miR-614	0.044	−1.74	6.489
hsa-miR-1234-3p	0.042	−1.73	6.385
hsa-miR-199b-5p	0.037	−1.72	5.89

P-value = Student's *t* test. Statistically significant FDR≤10%

Abbreviations: FC=Fold Change; FDR=False Discovery Rate.

### Survival analysis

Multivariate Cox regression analysis including clinical covariates and serum expression levels of the 18 differentially expressed miRNAs showed that miR-151b and pathological stage were significant prognostic factors for tumour progression (HR=0.33; p<0.001 and HR=2.62; p=0.006, respectively) and cancer specific survival (HR=0.25; p<0.001 and HR=3.98; p=0.003, respectively).

**Figure 2:**
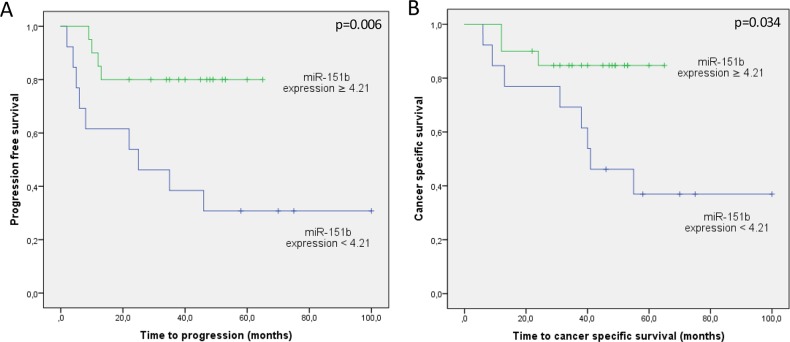
Kaplan Meier curves for **(A)** tumour progression and **(B)** cancer-specific survival according to miR-151b expression values (N=33). Green line represents patients at low risk (expression cutoff≥4.21) and blue line represents patients at high risk (expression cutoff<4.21).

Thereafter, the median expression value of miR-151b was used as a cut-off point to classify patients into a high-risk group (61%) and a low-risk group (39%) for tumour progression. Figure 2 depicts Kaplan-Meier curves generated using the selected cut-off point. As shown, the miR-151b expression value was able to discriminate between two groups of UTUC patients with a highly significant different probability of tumour progression (p=0.006) and cancer specific survival (p=0.034).

### Pathway analysis

**Figure 3:**
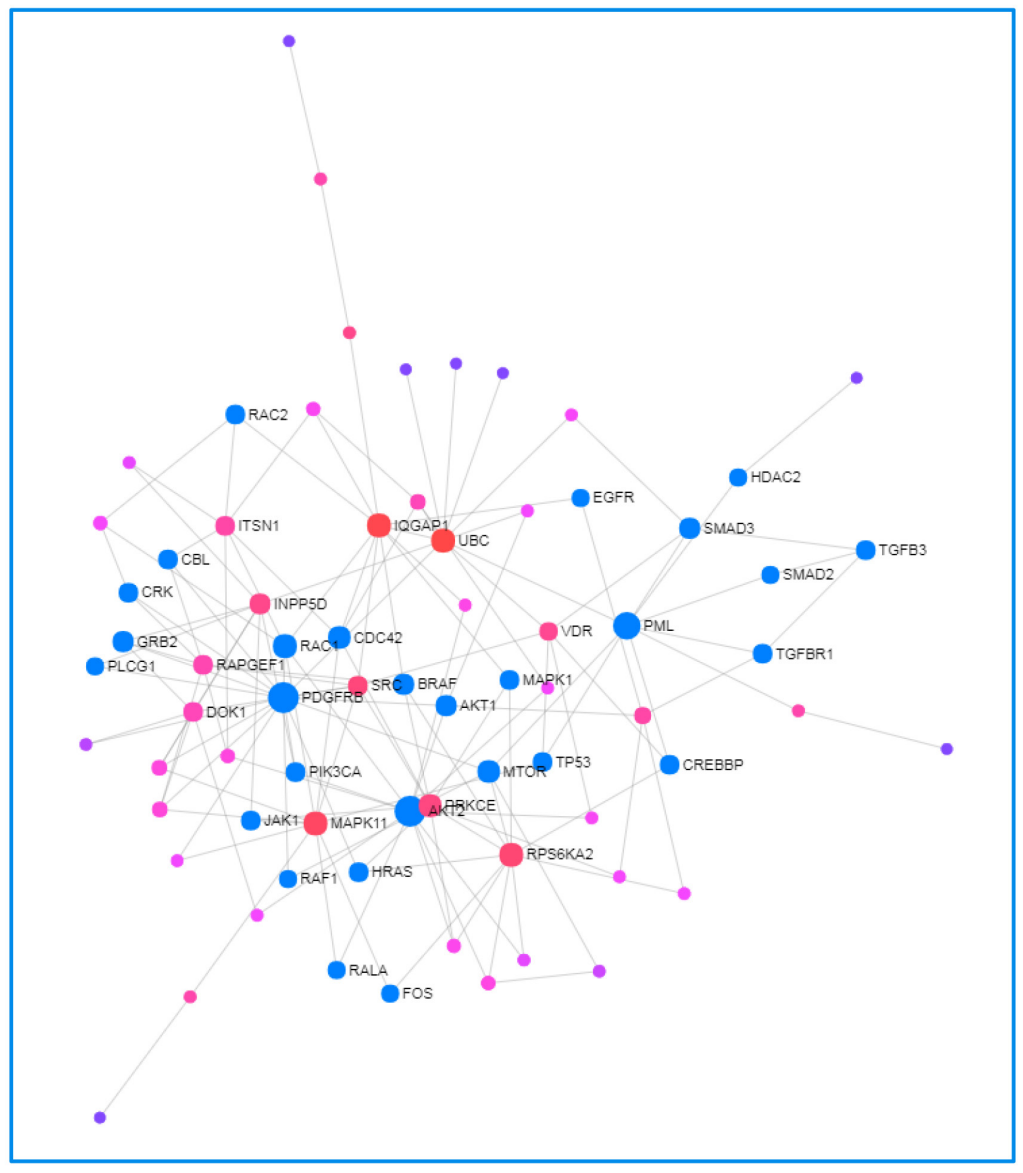
Network Analyst-derived protein–protein interactions network results Network constructed with the 53 genes shared across most of data sets as seed proteins. Red nodes are most important interactions, followed by pink nodes and finally purple nodes. Nodes in blue represent those proteins interacting in Cancer pathways.

Ingenuity® Pathway Analysis (IPA®) software predicted 104 target genes for miR-151b (Supplementary Table 1) while miRWalk analysis identified 1063 seed sequences (corresponding to 368 genes). There were 53 predicted gene targets for miR-151b with a statistically significant relation identified in common for both softwares (Supplementary Figure 1). Afterwards, Network Analyst showed several of these predicted targets to be related to Cancer pathways (Figure 3).

## DISCUSSION

UTUC is considered to have an unpredictable prognosis. In the present series, more than one third of the patients developed tumour progression and died from their cancer within four years of follow-up. Pathological stage and histological grade are the most commonly used prognostic factors for UTUC, but they are insufficient to accurately predict tumour progression. The identification of minimally invasive prognostic UTUC biomarkers would help to accurately estimate the progression risk at the time of diagnosis, and consequently to identify the most appropriate therapy for each individual patient. Furthermore, it may be useful for monitoring tumour progression after RNU.

Our group has prior experience in miRNA analysis from different types of urothelial cell carcinoma samples. On the one hand, we provided evidence that miRNAs can be used as prognostic biomarkers in UTUC in tissue samples [3, 4]. On the other, we showed that miRNAs can be useful diagnostic and prognostic biomarkers for bladder cancer in urine samples [12, 18]. Here, we evaluate circulating miRNA in the bloodstream from UTUC patients as minimally invasive predictive biomarkers of tumour progression and patient survival.

It has been previously described that circulating miRNAs in the bloodstream may be used as disease markers due to their methodological advantages over gene expression assays. First, it is easier to obtain short mature serum miRNAs than long-chain serum mRNA, since they are more stable against nuclease degradation [19]. In addition, the average copy number of an individual miRNA has been estimated to be ~ 500 per cell, which may be higher than the average mRNA species [20]. Consequently, less total RNA is required for a miRNA than for an mRNA assay. Regarding the technology used to quantify miRNA expression, here we have used nCounter (NanoString Technologies). This system is a simple and cost-effective methodology that allows gene expression quantification in a multiplex format. In addition, it does not need any nucleic acid amplification and it provides more accurate miRNA quantification than PCR or microarray-based methods, especially in samples with low abundant and degraded RNA [21–23]. Actually, this system is already used in clinical settings for diagnostic as well as prognostic purpouses [24, 25].

Therefore, circulating miRNAs may be a potentially useful blood-based tool for monitoring human cancers or predicting tumour outcome. In the case of urothelial carcinoma, some diagnostic miRNAs have been identified in serum samples from bladder cancer patients [26, 27]. Jiang X et al [28] evaluated miRNA expression in serum samples from muscle-invasive bladder tumour, and they found two miRNAs (miR-486-3p and miR-103a-3p) with prognostic value in these patients. In the case of UTUC, there are only few studies evaluating UTUC circulating miRNAs, and all of them have been focused on identifying diagnostic biomarkers. Kriebel *et al*., reported alterations in miRNA expression, both in serum (n=44) and tissue (n=47) samples from UTUC patients. They showed that circulating miR-141 may be useful as diagnostic biomarker [29]. Tao *et al*. using 46 serum samples from UTUC patients and 30 controls identified a tenserum miRNA-based expression profile that was useful in discriminating between UTUC cases and controls [17]. However, as far as we could ascertain, this is the first study that shows differences in the miRNA expression profile between serum samples from progressing and non-progressing UTUC patients and identifies a prognostic circulating miRNA (miR-151b) that independently predicts tumour progression and cancer specific survival. Unfortunately, neither the diagnostic nor prognostic serum miRNAs identified in bladder cancer samples are shared with miRNAs found in serum UTUC samples, supporting the previous genetic differences found in both tumour types [30, 31].

In accordance with our results, down-regulation of circulating miR-151b is described in primary CNS lymphoma patients [32]. Furthermore, *in silico* analysis shows that one of the target genes of miR-151b, *CCNE1,* is involved in T-cell signaling, cell-cycle, DNA-damage induced signaling and breast, pancreas, lung and prostate cancer pathways. Interestingly, *CCNE1* has been previously suggested as a prognostic biomarker for UTUC [33]. Wu S *et al*. [34] demonstrated that over-expression of *CCNE1* is associated with lower overall survival in UTUC patients, which is in concordance with our results showing down-regulation of miR-151b in progressing patients.

We are aware, however, that the study has some limitations. First, the total number of patients analyzed can be considered as low. It should be taken into account that UTUC is a rare disease, accounting only for 5-10% of all urothelial carcinomas, which makes it difficult to obtain large prospective series. Furthermore, we followed a very restrictive inclusion criterion, excluding all those patients having UTUC and other active neoplasms. Second, miRNA isolation from serum samples is challenging since the amount of circulating miRNA is low. Even so, we were able to analyze miRNA expression in all samples except two (informative specimen rate of 94%). Finally, the data reported warrants further prospective evaluation in carefully and specifically designed studies.

In conclusion, our results demonstrate that serum samples from progressive and non-progressive UTUC patients show a differential miRNA expression pattern. We also show that expression of miRNA-151b in serum samples significantly predicts progression and cancer-specific survival in UTUC patients, indicating that it may be a novel potential minimally invasive biomarker for prognosis of UTUC patients. Although independent validation of the data is necessary, the identification of new circulating miRNAs associated with poor outcome in patients with UTUC may help tailor treatment and surveillance strategies in these patients.

## MATERIALS AND METHODS

### Patients and clinical samples

Prospective study including 35 UTUC patients who underwent radical RNU at the Hospital Clinic of Barcelona from January 2008 to August 2014. Finally, 33 UTUC patients (12 progressing and 21 non-progressing) were analyzed. Consecutive patients with a minimum of 36 months of follow-up were enrolled. Exclusion criteria were presence of another active neoplasm or absence of UTUC confirmation. Patients' histopathological characteristics are summarized in Table 2. Histological Grade and Pathological Stage of the tumors were determined according to WHO criteria and TNM classification, respectively [35, 36]. Institutional Review Board approval and patients' informed consents were obtained from all patients.

**Table 2:** Pathological features of UTUC patients

	Progressing UTUC N (%)	Non-Progressing UTUC N (%)	Total N (%)
**N° Patients**	**13**	**20**	**33**
**Gender**			
Male	8 (62)	15 (75)	**23 (70)**
Female	5 (38)	5 (25)	**10 (30)**
**Tumor location**			
Pelvis	5	14	**19 (58)**
Ureter	7	5	**12 (36)**
Both	1	1	**2 (6)**
**Pathological Stage**			
pTa	1 (8)	6 (30)	**7 (21)**
pT1	1 (8)	4 (20)	**5 (15)**
pT2	3 (23)	4 (20)	**7 (21)**
pT3	6 (46)	6 (30)	**12 (37)**
pT4	2 (15)	0 (0)	**2 (6)**
**Histological Grade**			
Low	2 (15)	4 (20)	**6 (18)**
High	11 (85)	16 (80)	**27 (82)**
**Metastasis**			
Local	3 (24)	-	3 (24)
Distant	5 (37)	-	5 (37)
Local + Distant	2 (15)	-	2 (15)
Nodes	3 (24)	-	3 (24)
**Chemotherapy**			
Adjuvant	9 (69)	-	9 (69)

All patients were diagnosed by computed tomography (CT) scan and followed-up postoperatively with CT scan and cystoscopy at 3-month intervals for the first year, at 6-month intervals for the next 2 years, and annually thereafter. Tumours were considered as progressing when distant metastasis or pathological nodes were developed during follow-up.

This study was split into a two-stage approach with an initial discovery phase (training set) and a validation phase (testing set). Initial discovery phase included 12 UTUC patients, 5 progressing [1 pT2, 3 pT3 and 1 pT4, all high grade (HG)] and 7 non-progressing (2 pTa, 1 pT2 and 4 pT3, all HG). Validation phase comprised 21 UTUC patients, 8 progressing [1 pTa low grade (LG), 1 pT1 LG, 2 pT2 HG, 3 pT3 HG and 1 pT4 HG] and 13 non-progressing (4 pTa LG, 4 pT1 HG, 3 pT2 HG and 2 pT3 HG).

### Serum sample processing and RNA isolation

Whole blood samples were collected in the operating room before RNU in a BD Vacutainer 10mL sterile tube coated with silicone and micronized silica particles and stored at 4°C. Blood was left to clot for a minimun of one hour and, within four hours, tubes were centrifuged for 15 minutes at 3500rpm, 4°C. Serum was immediately transferred to a cryotube and stored in aliquots at −80°C until use.

Total RNA was isolated from 500μL of serum aliquots using mirVana PARIS Kit (Thermosfisher Scientific) according to the manufacturer's instructions. Before RNA isolation, 5μL at 200pM of five Spike-In controls (ath-miR-159a, cel-miR-248, cel-miR-254, osa-miR-414 and osa-miR-442) were added to each sample. Total RNA was quantified with a NanoDrop1000 (NanoDrop Technologies, Wilmington, DE, USA).

### Expression analysis

Expression profiling of 800 human miRNAs from miRBase v3 was analyzed with the nCounter Human v3 miRNA Expression Assay Kit (NanoString Technologies) following manufacturer's instructions. Briefly, the assay uses molecular barcodes called nCounter Reporter Probes to detect microRNAs. The reaction involves a multiplexed hybridization of the specific probes to their target miRNA and an enzymatic purification to remove the unligated probes on the nCounter Prep Station. Data is finally collected by direct digital counting of the target molecules in each sample using the nCounter Digital analyzer. The assay contains positive and negative controls, reference genes and spike-in controls.

Five spike-in controls were used to compensate variations in RNA recovery between samples. Six established housekeeping miRNAs for serum already included in the assay (hsa-miR-16-5p, hsa-miR-484, hsa-miR-126-3p, hsa-miR-191-5p, hsa-miR-93-5p and hsa-miR-24-3p) were used to normalize microRNA expression levels [37–39]. After normalization, fold change expression between progressing and non-progressing patients was calculated using the moderate p-value (FDR) of the limma-R package [40].

### Statistical analysis

Univariate Cox regression analysis was performed on the established clinical prognosticators of UTUC (stage and grade) and the 18 differentially expressed miRNAs to examine its influence on tumor progression and cancer specific survival; afterwards, multivariate forward stepwise Cox regression analysis was performed on significant covariates. Statistical significance was established at a p-value of 0.05 and according to 95% confidence intervals (CI). miRNA expression was dichotomized using the median expression value of miR-151b (cutoff = 4.21). Thereafter, Kaplan-Meier curves were generated. SPSS 23.0 software was used for statistical analysis.

### Pathway enrichment analysis

The biological targets of the miRNAs identified were investigated using IPA®. Interactions and networks between significant miRNAs and genes were mapped to pathways, regulators, diseases, and functions based on direct/indirect and experimentally validated targets.

The miRWalk software [41] was used to predict putative miRNA binding sites in target genes, in order to discover possible canonical altered pathways. Finally, Network Analyst [42] was used to determine protein-protein interactions of the resulting genes from the previous analysis.

## SUPPLEMENTARY MATERIALS FIGURE AND TABLE





## Abbreviations

RNU: Radical Nephroureterectomy
UTUC: Upper Tract Urothelial Carcinoma
CI: confidence interval
CT scan: computed tomography scan
LG: low grade
HG: high grade
HR: hazard ratio
IPA: Ingenuity Pathway Analysis
miRNA: microRNA

## References

[1] Roupret M, Zigeuner R, Palou J, Boehle A, Kaasinen E, Sylvester R, Babjuk M, Oosterlinck W (2011). European guidelines for the diagnosis and management of upper urinary tract urothelial cell carcinomas: 2011 update. Eur Urol.

[2] Huben RP, Mounzer AM, Murphy GP (1988). Tumor grade and stage as prognostic variables in upper tract urothelial tumors. Cancer.

[3] Izquierdo L, Ingelmo-Torres M, Mallofre C, Lozano JJ, Verhasselt-Crinquette M, Leroy X, Colin P, Comperat E, Roupret M, Alcaraz A, Mengual L (2014). Prognostic value of microRNA expression pattern in upper tract urothelial carcinoma. BJU Int.

[4] Izquierdo L, Montalbo R, Ingelmo-Torres M, Mallofre C, Ramirez-Backhaus M, Rubio J, Van der Heijden AG, Schaafsma E, Lopez-Beltran A, Blanca A, Lawrentschuk N, Alcaraz A, Mengual L (2017). Prognostic microRNAs in upper tract urothelial carcinoma: multicenter and international validation study. Oncotarget.

[5] Popovska-Jankovic K, Noveski P, Jankovic-Velickovic L, Stojnev S, Cukuranovic R, Stefanovic V, Toncheva D, Staneva R, Polenakovic M, Plaseska-Karanfilska D (2016). MicroRNA profiling in patients with upper tract urothelial carcinoma associated with balkan endemic nephropathy. Biomed Res Int.

[6] Ke HL, Li WM, Lin HH, Hsu WC, Hsu YL, Chang LL, Huang CN, Li CC, Chang HP, Yeh HC, Li CF, Wu WJ (2017). Hypoxia-regulated microRNA-210 overexpression is associated with tumor development and progression in upper tract urothelial carcinoma. Int J Med Sci.

[7] Zhang BH, Pan XP, Wang QL, Cobb GP, Anderson TA (2005). Identification and characterization of new plant microrNAS using EST analysis. Cell Res.

[8] Lin T, Dong W, Huang J, Pan Q, Fan X, Zhang C, Huang L (2009). MicroRNA-143 as a tumor suppressor for bladder cancer. J Urol.

[9] Vogt M, Munding J, Gruner M, Liffers ST, Verdoodt B, Hauk J, Steinstraesser L, Tannapfel A, Hermeking H (2011). Frequent concomitant inactivation of miR-34a and miR-34b/c by CpG methylation in colorectal, pancreatic, mammary, ovarian, urothelial, and renal cell carcinomas and soft tissue sarcomas. Virchows Arch.

[10] Yamada Y, Enokida H, Kojima S, Kawakami K, Chiyomaru T, Tatarano S, Yoshino H, Kawahara K, Nishiyama K, Seki N, Nakagawa M (2011). MiR-96 and miR-183 detection in urine serve as potential tumor markers of urothelial carcinoma: correlation with stage and grade, and comparison with urinary cytology. Cancer Sci.

[11] Catto JW, Miah S, Owen HC, Bryant H, Myers K, Dudziec E, Larre S, Milo M, Rehman I, Rosario DJ, Di Martino E, Knowles MA, Meuth M (2009). Distinct microRNA alterations characterize high- and low-grade bladder cancer. Cancer Res.

[12] Mengual L, Lozano JJ, Ingelmo-Torres M, Gazquez C, Ribal MJ, Alcaraz A (2013). Using microRNA profiling in urine samples to develop a non-invasive test for bladder cancer. Int J Cancer.

[13] Chen X, Ba Y, Ma L, Cai X, Yin Y, Wang K, Guo J, Zhang Y, Chen J, Guo X, Li Q, Li X, Wang W (2008). Characterization of microRNAs in serum: a novel class of biomarkers for diagnosis of cancer and other diseases. Cell Res.

[14] Mitchell PS, Parkin RK, Kroh EM, Fritz BR, Wyman SK, Pogosova-Agadjanyan EL, Peterson A, Noteboom J, O'Briant KC, Allen A, Lin DW, Urban N, Drescher CW (2008). Circulating microRNAs as stable blood-based markers for cancer detection. Proc Natl Acad Sci U S A.

[15] Zuo Z, Maiti S, Hu S, Loghavi S, Calin GA, Garcia-Manero G, Kantarjian HM, Medeiros LJ, Cooper LJ, Bueso-Ramos CE (2015). Plasma circulating-microRNA profiles are useful for assessing prognosis in patients with cytogenetically normal myelodysplastic syndromes. Mod Pathol.

[16] Huang X, Liang M, Dittmar R, Wang L (2013). Extracellular micrornas in urologic malignancies: chances and challenges. Int J Mol Sci.

[17] Tao J, Yang X, Li P, Wei J, Deng X, Cheng Y, Qin C, Ju X, Meng X, Li J, Gu M, Lu Q, Yin C (2015). Identification of circulating microRNA signatures for upper tract urothelial carcinoma detection. Mol Med Rep.

[18] Ingelmo-Torres M, Lozano JJ, Izquierdo L, Carrion A, Costa M, Gomez L, Ribal MJ, Alcaraz A, Mengual L (2017). Urinary cell microRNA-based prognostic classifier for non-muscle invasive bladder cancer. Oncotarget.

[19] Cortez MA, Bueso-Ramos C, Ferdin J, Lopez-Berestein G, Sood AK, Calin GA (2011). MicroRNAs in body fluids--the mix of hormones and biomarkers. Nat Rev Clin Oncol.

[20] Ragan C, Zuker M, Ragan MA (2011). Quantitative prediction of miRNA-mRNA interaction based on equilibrium concentrations. PLoS Comput Biol.

[21] Geiss GK, Bumgarner RE, Birditt B, Dahl T, Dowidar N, Dunaway DL, Fell HP, Ferree S, George RD, Grogan T, James JJ, Maysuria M, Mitton JD (2008). Direct multiplexed measurement of gene expression with color-coded probe pairs. Nat Biotechnol.

[22] Reis PP, Waldron L, Goswami RS, Xu W, Xuan Y, Perez-Ordonez B, Gullane P, Irish J, Jurisica I, Kamel-Reid S (2011). mRNA transcript quantification in archival samples using multiplexed, color-coded probes. BMC Biotechnol.

[23] Wang H, Horbinski C, Wu H, Liu Y, Sheng S, Liu J, Weiss H, Stromberg AJ, Wang C (2016). Nanostringdiff: a novel statistical method for differential expression analysis based on nanostring ncounter data. Nucleic Acids Res.

[24] Prat A, Galvan P, Jimenez B, Buckingham W, Jeiranian HA, Schaper C, Vidal M, Alvarez M, Diaz S, Ellis C, Nuciforo P, Ferree S, Ribelles N (2016). Prediction of response to neoadjuvant chemotherapy using core needle biopsy samples with the prosigna assay. Clin Cancer Res.

[25] Martin M, Gonzalez-Rivera M, Morales S, Haba-Rodriguez J, Gonzalez-Cortijo L, Manso L, Albanell J, Gonzalez-Martin A, Gonzalez S, Arcusa A, Cruz-Merino L, Rojo F, Vidal M (2015). Prospective study of the impact of the prosigna assay on adjuvant clinical decision-making in unselected patients with estrogen receptor positive, human epidermal growth factor receptor negative, node negative early-stage breast cancer. Curr Med Res Opin.

[26] Jiang X, Du L, Wang L, Li J, Liu Y, Zheng G, Qu A, Zhang X, Pan H, Yang Y, Wang C (2015). Serum microrna expression signatures identified from genome-wide microrna profiling serve as novel noninvasive biomarkers for diagnosis and recurrence of bladder cancer. Int J Cancer.

[27] Motawi TK, Rizk SM, Ibrahim TM, Ibrahim IA (2016). Circulating microRNAs, miR-92a, miR-100 and miR-143, as non-invasive biomarkers for bladder cancer diagnosis. Cell Biochem Funct.

[28] Jiang X, Du L, Duan W, Wang R, Yan K, Wang L, Li J, Zheng G, Zhang X, Yang Y, Wang C (2016). Serum microrna expression signatures as novel noninvasive biomarkers for prediction and prognosis of muscle-invasive bladder cancer. Oncotarget.

[29] Kriebel S, Schmidt D, Holdenrieder S, Goltz D, Kristiansen G, Moritz R, Fisang C, Muller SC, Ellinger J (2015). Analysis of tissue and serum microRNA expression in patients with upper urinary tract urothelial cancer. PLoS One.

[30] Moss TJ, Qi Y, Xi L, Peng B, Kim TB, Ezzedine NE, Mosqueda ME, Guo CC, Czerniak BA, Ittmann M, Wheeler DA, Lerner SP, Matin SF (2017). Comprehensive genomic characterization of upper tract urothelial carcinoma. Eur Urol.

[31] Izquierdo L, Mengual L, Gazquez C, Ingelmo-Torres M, Alcaraz A (2009). Molecular characterization of upper urinary tract tumours. BJU Int.

[32] Roth P, Keller A, Hoheisel JD, Codo P, Bauer AS, Backes C, Leidinger P, Meese E, Thiel E, Korfel A, Weller M (2015). Differentially regulated mirnas as prognostic biomarkers in the blood of primary cns lymphoma patients. Eur J Cancer.

[33] Wu S, Chen J, Dong P, Zhang S, He Y, Sun L, Zhu J, Cheng Y, Li X, Tang A, Huang Y, Gui Y, Liu C (2014). Global gene expression profiling identifies ALDH2, CCNE1 and SMAD3 as potential prognostic markers in upper tract urothelial carcinoma. BMC Cancer.

[34] Guo B, Luo C, Xun C, Xie J, Wu X, Pu J (2009). Quantitative detection of cytokeratin 20 mRNA in urine samples as diagnostic tools for bladder cancer by real-time PCR. Exp Oncol.

[35] Lopez-Beltran A, Sauter G, Gasser T, Hartmann A, Schmitz-Dräger BJ, Helpap B, Ayala AG, Tamboni P, Knowles MA, Sidransky D, Cordon-Cardo C, Jones PA, Cairns P, World Health Organization (2004). Classification of Tumours. Pathology and Genetics. Tumours of the Urinary System and Male Genital Organs.

[36] Sobin LH, Wittekind CH (2002). TNM Classification of Malignant Tumours.

[37] Marabita F, de Candia P, Torri A, Tegner J, Abrignani S, Rossi RL (2016). Normalization of circulating microrna expression data obtained by quantitative real-time RT-PCR. Brief Bioinform.

[38] Song J, Bai Z, Han W, Zhang J, Meng H, Bi J, Ma X, Han S, Zhang Z (2012). Identification of suitable reference genes for qPCR analysis of serum microRNA in gastric cancer patients. Dig Dis Sci.

[39] Hu Z, Dong J, Wang LE, Ma H, Liu J, Zhao Y, Tang J, Chen X, Dai J, Wei Q, Zhang C, Shen H (2012). Serum microRNA profiling and breast cancer risk: the use of miR-484/191 as endogenous controls. Carcinogenesis.

[40] Ritchie ME, Phipson B, Wu D, Hu Y, Law CW, Shi W, Smyth GK (2015). Limma powers differential expression analyses for RNA-sequencing and microarray studies. Nucleic Acids Res.

[41] Dweep H, Sticht C, Pandey P, Gretz N (2011). Mirwalk--database: prediction of possible mirna binding sites by “walking” the genes of three genomes. J Biomed Inform.

[42] Xia J, Gill EE, Hancock RE (2015). Networkanalyst for statistical, visual and network-based meta-analysis of gene expression data. Nat Protoc.

