# Supplementary material for: Prognostic value of circulating microRNAs in upper tract urinary carcinoma
Source: Oncotarget. 2018 Mar 30;9(24):16691–700. doi: 10.18632/oncotarget.24672 (PMC5908279; doi:10.18632/oncotarget.24672)
Supplement: Supplementary file 1 [file oncotarget-09-16691-s001.pdf]

Prognostic value of circulating microRNAs in upper tract urinary carcinoma

SUPPLEMENTARY MATERIALS

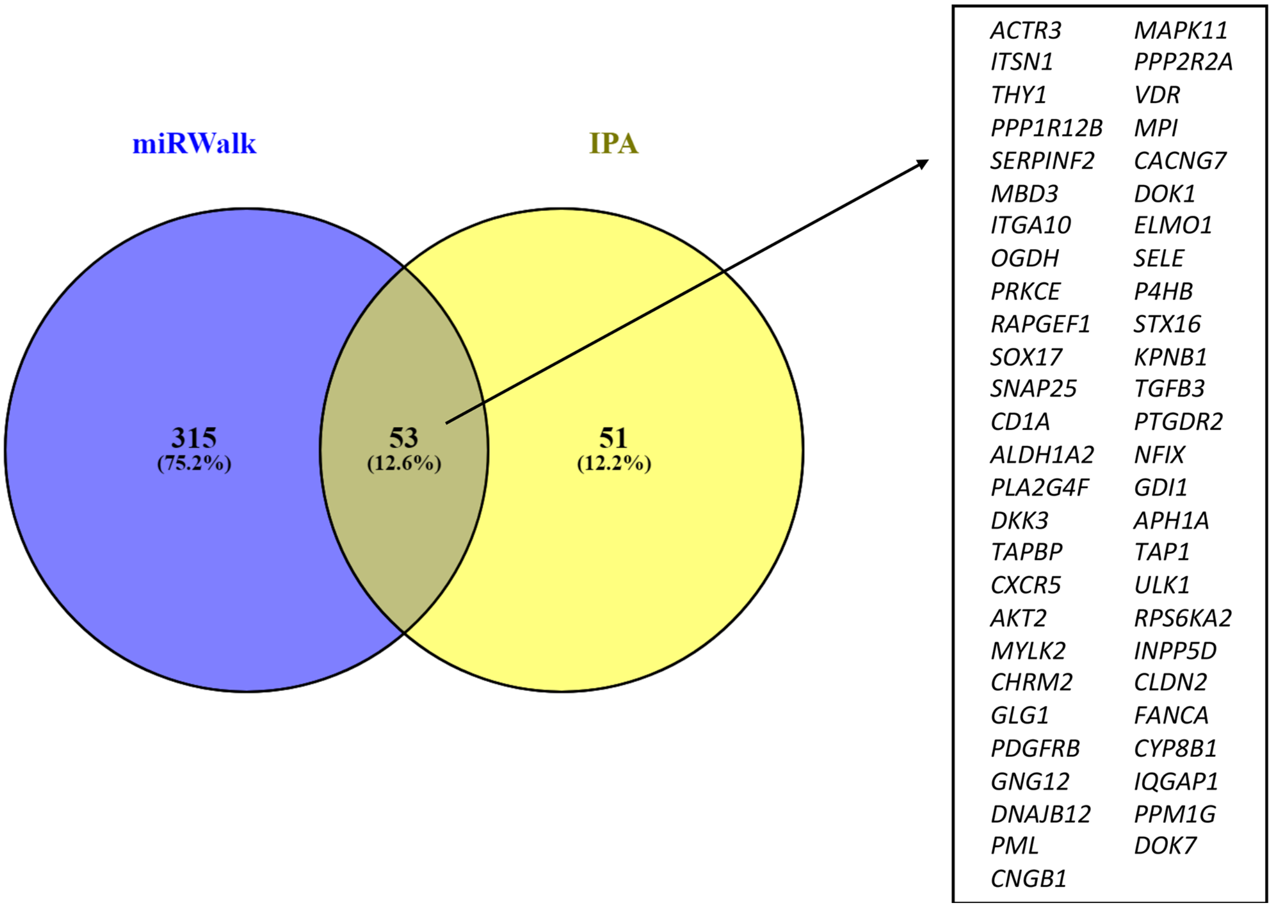

Supplementary Figure 1: Venny diagram showing target genes for miR-151b from miRWalk and IPA analysis. The 53 genes in common are listed in the box.

Supplementary Table 1: IPA predicted gene targets for miR-151b using TagetScan Human source.

See Supplementary File 1
